# Discrete subaortic stenosis: prognostic value of the left ventricular outflow tract gradient and implications for earlier surgical intervention

**DOI:** 10.1007/s12519-026-01048-z

**Published:** 2026-06-11

**Authors:** Yu-Tong Jiang, Ting-Ting Zhang, Li Zhang, Nan Xu, Bing Zhang, Jia-Qi Zhang, Ke-Ming Yang, Shou-Jun Li, Kun-Jing Pang

**Affiliations:** 1 Department of Echocardiography, National Center for Cardiovascular Disease, Fuwai Hospital, Chinese Academy of Medical Sciences and Peking Union Medical College, Beijing, China; 2 Pediatric Cardiac Surgery Center, Fuwai Hospital, National Center for Cardiovascular Disease, Chinese Academy of Medical Sciences and Peking Union Medical College, Beijing, China

**Keywords:** Aortic valve stenosis, Congenital heart defects, Echocardiography, Risk assessment

## Abstract

**Background:**

The optimal timing of surgery for discrete subaortic stenosis (DSS) is controversial due to limited evidence from large-scale trials. This study aimed to identify prognostic factors and reassess current surgical thresholds.

**Methods:**

A total of 508 patients with DSS, including 375 pediatric patients, who underwent surgery at a single tertiary center between May 2018 and March 2025 were retrospectively analyzed. The primary endpoint was a composite of DSS-related adverse events, and the secondary endpoint was aortic valve (AV) dysfunction. Multivariable Cox regression and receiver operating characteristic (ROC) analyses were used to identify predictors.

**Results:**

Over a mean follow-up period of 3.5 years, 19.2% of pediatric patients experienced the primary endpoint and 13.3% developed AV dysfunction. Left ventricular outflow tract gradients (LVOTG) ≥ 20 mmHg [hazards ratio (HR) = 4.0, 95% confidence interval (CI): 2.0–7.8, *P* < 0.001], ≥ 35 mmHg (HR = 6.1, 95% CI: 3.2–11.6, *P* < 0.001), and ≥ 50 mmHg (HR = 3.5, 95% CI: 1.9–6.4, *P* < 0.001) were all independently associated with adverse outcomes. Of note, an LVOTG ≥ 35 mmHg provided comparable prognostic accuracy to ≥ 50 mmHg (*P* = 0.180) but superior risk discrimination compared with 20 mmHg ≤ LVOTG < 35 mmHg (*P* < 0.001). Additional independent predictors included older age at surgery, moderate or greater aortic regurgitation.

**Conclusions:**

An LVOTG threshold of ≥ 35 mmHg may offer a more appropriate risk-alert threshold than the current ≥ 50 mmHg standard. Earlier surgical intervention, particularly in patients with AV involvement, may help improve long-term outcomes in DSS.

**Graphical abstract:**

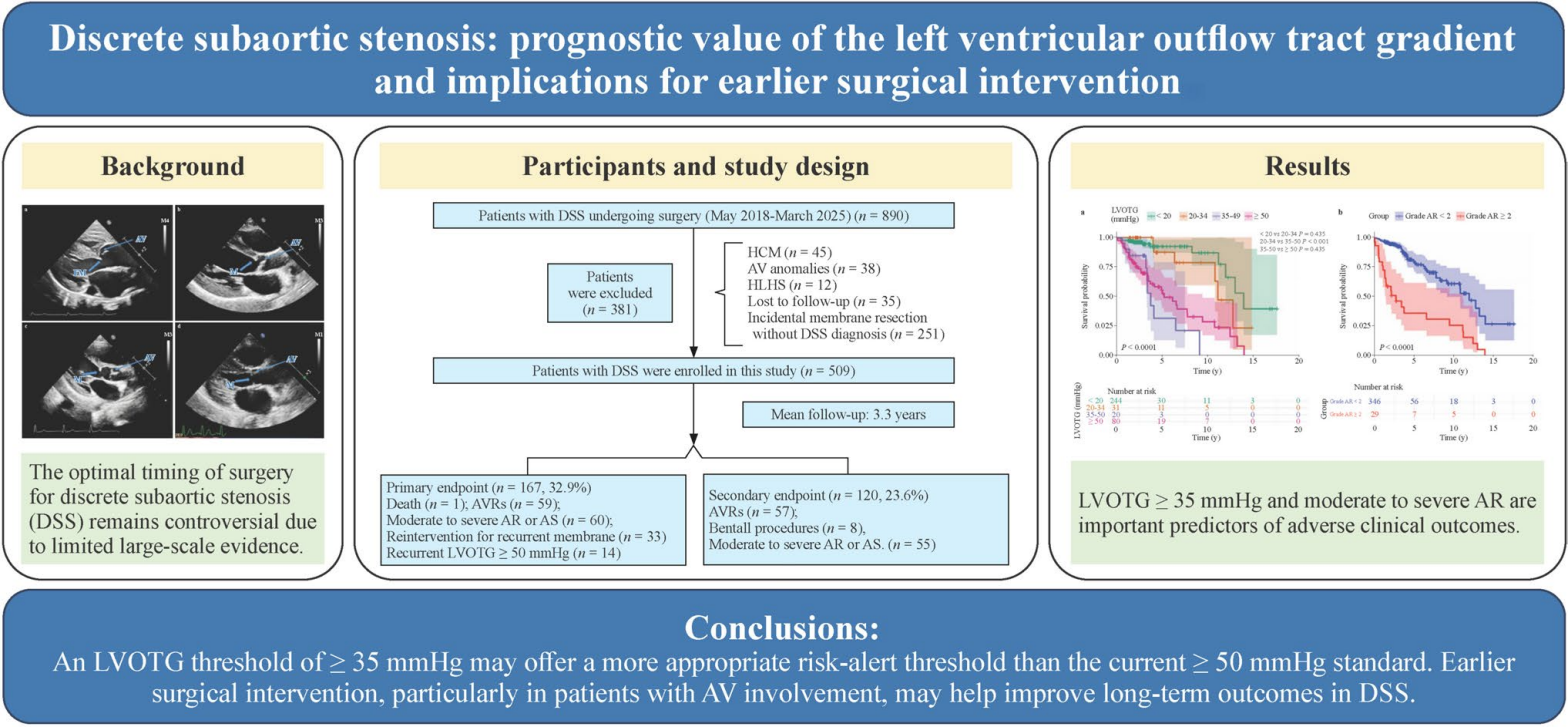

**Supplementary Information:**

The online version contains supplementary material available at 10.1007/s12519-026-01048-z.

## Introduction

Discrete subaortic stenosis (DSS) is encountered with appreciable frequency in both pediatric and adult congenital heart disease populations [1, 2]. The principal hemodynamic disturbances, left ventricular outflow tract obstruction and aortic valve (AV) dysfunction, are associated with adverse outcomes and may compromise survival [3–8]. However, the optimal timing and indications for surgical intervention in DSS are controversial, in part because of scarce data from large cohort studies. At most centers, current indications for surgery are: (1) left ventricular outflow tract gradients (LVOTG) exceeding 50 mmHg estimated by echocardiography; or (2) moderate or greater aortic regurgitation, for which surgery may be considered irrespective of gradient severity [6, 7]. It is unclear whether adhering to these criteria yields the best clinical outcomes, such as preventing recurrent left ventricular outflow tract (LVOT) obstruction necessitating reoperation or preserving native AV function to avoid prosthetic replacement. To address this gap, we conducted a retrospective analysis of DSS patients who underwent surgery at our center, with the dual aims of identifying predictors of adverse postoperative outcomes and assessing whether existing surgical indications merit revision.

## Methods

### Patient population

This retrospective study included consecutive patients with DSS who underwent surgical intervention at Fuwai Hospital between May 2018 and March 2025. The patient selection process is illustrated in Supplementary Fig. 1. Patients were excluded if they had hypertrophic cardiomyopathy (*n* = 45), aortic valve anomalies (*n* = 38), hypoplastic left heart syndrome (*n* = 12), or were lost to follow-up (*n* = 35). In addition, we excluded patients without preoperative evidence of LVOT flow acceleration on echocardiography or without a documented preoperative echocardiographic diagnosis of DSS, in whom a short subaortic membrane was incidentally resected during surgery for other congenital heart anomalies (*n* = 251). Baseline clinical and operative data were extracted from electronic medical records. All patients underwent preoperative transthoracic two-dimensional echocardiography; imaging data were archived digitally for subsequent analysis. The study protocol was approved by the Ethics Committee of Fuwai Hospital (Approval No. 2022–1750), and informed consent was waived due to the retrospective nature of the study and anonymized data. The study was conducted in accordance with the Strengthening the Reporting of Observational Studies in Epidemiology guidelines.

### Diagnosis and typology of discrete subaortic stenosis

**Fig. 1:**
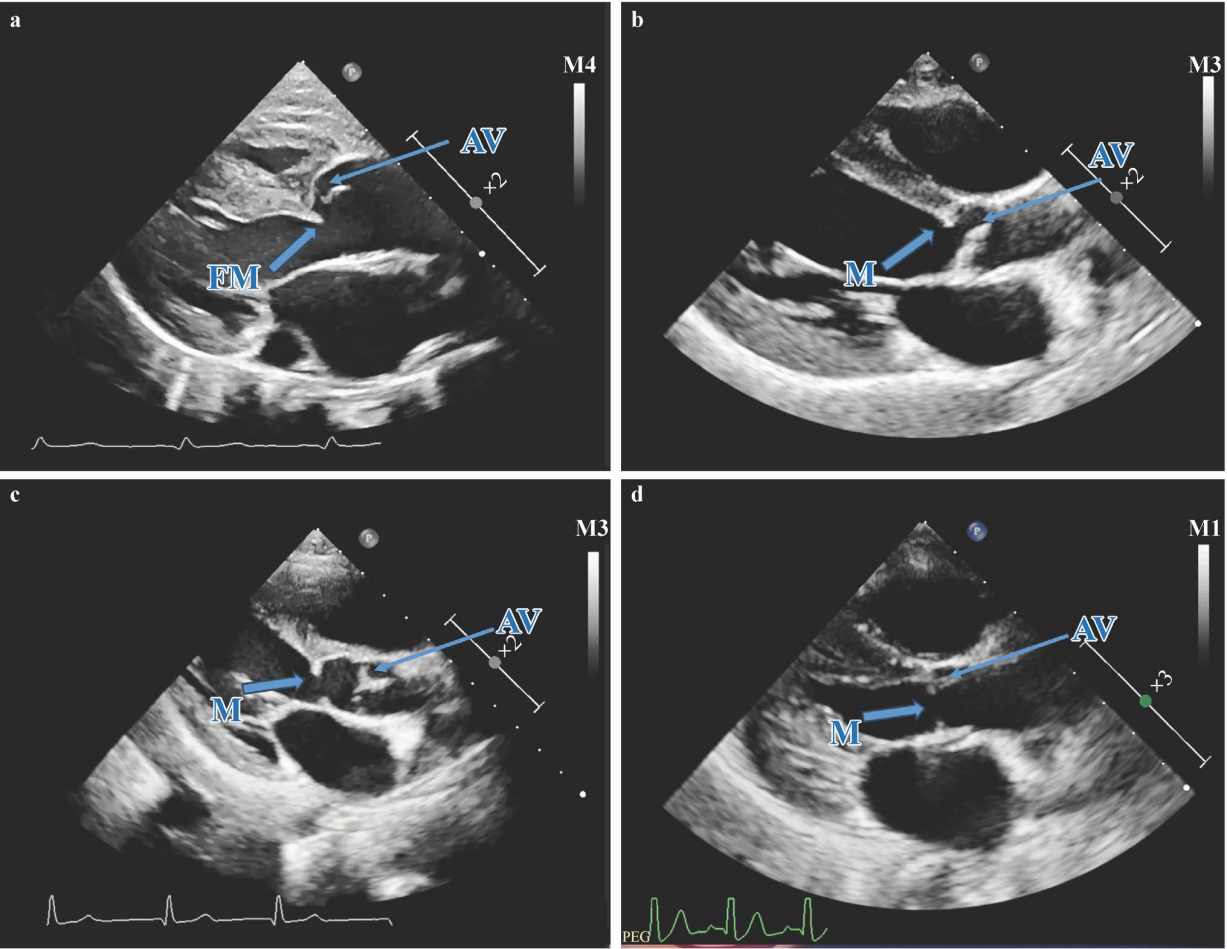
Four subtypes of DSS patients. **a** Semilunar membrane, located at a distance from the AV; **b** semilunar membrane, attached to the AV; **c** circumferential membrane, located at a distance from the AV; **d** circumferential membrane, attached to the AV. The thick blue arrow indicates the subaortic membrane, while the thin blue arrow points to the AV. *DSS* discrete subaortic stenosis, *AV* aortic valve, *FM* fibromuscular ridge type of membrane, *M* membranous type

DSS was initially diagnosed by echocardiography and was based on the presence of a subaortic membrane or fibromuscular ridge [7, 9] (Fig. 1). The anatomical subtype and diameter of the lesion were confirmed intraoperatively. DSS was classified into two subtypes: membrane and fibromuscular ridge. Membrane morphology was further categorized as circumferential or crescent-shaped. Membrane attachment to the AV was documented separately [3, 7].

### Surgical indications

Surgical indications for DSS are as follows. (1) DSS with a significant gradient: echocardiography-measured LVOTG ≥ 50 mmHg; (2) DSS with an LVOTG < 50 mmHg accompanied by moderate to severe AV regurgitation, or DSS associated with other congenital cardiac anomalies requiring surgical correction, in which case DSS repair should be performed concomitantly.

### Follow-up and endpoints

Postoperative follow-up was conducted by clinical visits to assess membrane recurrence, LVOT obstruction and AV function. The primary endpoint was defined as a composite of adverse events related to DSS, including any of the following: (1) cardiac death; (2) prosthetic aortic valve replacement (AVR) or Bentall procedure performed when AV repair was deemed unfeasible or in the presence of a markedly dilated ascending aorta; (3) LVOTG ≥ 50 mmHg attributable to membrane recurrence; (4) surgical reintervention for DSS; or (5) AV dysfunction, defined as moderate or severe aortic stenosis (AS) or aortic regurgitation (AR). Patients who experienced any of the above outcomes were defined as the primary endpoints (+) group, while those who did not were defined as the primary endpoints (–) group. Membrane recurrence without a resulting LVOTG ≥ 50 mmHg and surgical reintervention unrelated to DSS were not recorded as adverse events. The secondary endpoint was defined as AV dysfunction, including AVR or development/progression to moderate or severe AS or AR. The index date (time zero) for follow-up was the date of the procedure (surgery or intervention). Early failures and deaths were retained in the analytic cohort and incorporated into endpoint assessments. Reoperations or adverse outcomes resulting solely from the correction of other cardiac lesions, such as residual or recurrent ventricular septal defect (VSD), patent ductus arteriosus and coarctation repair were explicitly excluded from the endpoint assessment.

### Statistical analysis

Categorical variables were displayed as frequencies (percentages). Continuous variables were displayed as means ± standard deviation or medians [interquartile ranges (IQR)], as appropriate. The normality of distribution was assessed by Kolmogorov–Smirnov tests, Q–Q plots and histograms. Comparisons between two groups were performed using the Chi-square test for categorical variables, Student’s t-test for normally distributed continuous variables and the Mann–Whitney *U* test for non-normally distributed continuous variables. Receiver operating characteristic (ROC) curve analysis was used to evaluate the predictive performance of age and LVOTG for the selected endpoints. Optimal cutoff was determined by maximizing the Youden Index. Comparisons of survival differences between groups were performed using Kaplan–Meier curve analysis along with the log-rank test. Univariable and multivariable Cox proportional hazards regression analyses were performed to identify risk factors for primary and secondary endpoints and reported with hazard ratios (HRs) and 95% confidence intervals (CIs). The proportional hazards assumption was assessed using Schoenfeld residuals with the cox. zph function from the survival package in R, both for each covariate and for the global model. To compare prognostic discrimination across LVOTG thresholds, we estimated time-dependent area under the ROC curves (AUCs) at 1, 3, and 5 years using inverse probability of censoring weighting. Pairwise differences in AUCs were quantified via 1000-bootstrap resampling to obtain ΔAUC, 95% CIs and *P* values. Multiple testing was controlled using the Holm procedure. As a sensitivity analysis, we also compared Uno’s C-index across models. Primary interpretation focused on the pediatric cohort because DSS is fundamentally a childhood disease, while the overall-cohort analysis was retained as a complementary analysis and adult findings were summarized secondarily. All statistical analyses were conducted using SPSS Statistics (version 26.0, IBM, SPSS Statistics) and R (version 4.3.2, R Foundation for Statistical Computing). A *P* value < 0.05 was considered statistically significant.

## Results

### Patient population

Because DSS lesions usually originate in childhood, the pediatric cohort (*n* = 375) was used as the main population for result presentation. Table 1 summarizes the baseline characteristics of pediatric patients according to primary endpoint occurrence. In the pediatric cohort, patients who reached the primary endpoint were older at the time of surgery, more likely to have New York Heart Association (NYHA) class > II, prior cardiac procedures, AR grade ≥ 2, membrane attachment to the AV and concomitant AV repair. In contrast, concomitant VSD and membrane-type DSS lesions were more common in those without primary endpoints. For completeness, the baseline characteristics of the entire cohort are presented in Supplementary Table 1. The cohort was formed by combining the original pediatric cohort with 133 (26.1%) additional adult patients; of these adults, 63 had residual DSS after prior simple congenital heart disease surgery at outside hospitals, whereas 70 had been diagnosed with DSS during childhood at other institutions but had not received treatment.

**Table 1:** Baseline characteristics according to primary endpoint (age < 18 years)

Variables	General, *N* = 375	Primary endpoint (–)^b^, *n* = 303	Primary endpoint (+)^a^, *n* = 72	*P*
Clinical characteristics				
Age (y)	3.3 (1.2, 6.1)	3.0 (1.1, 5.0)	8.6 (3.6, 12.3)	< 0.001
Male	212 (56.5)	175 (57.8)	37 (51.4)	0.327
BSA (m^2^)	0.6 (0.4, 0.9)	0.6 (0.4, 0.8)	1.0 (0.7, 1.4)	< 0.001
NYHA class ≥ II	51 (13.6)	28 (9.2)	23 (31.9)	< 0.001
History of cardiac procedures	53 (14.6)	26 (8.5)	27 (37.5)	< 0.001
History of DSS-related procedure	12 (3.2)	4 (1.3)	8 (11.1)	< 0.001
Concomitant cardiac malformations				
VSD	254 (67.7)	231 (76.2)	23 (31.9)	< 0.001
PDA	55 (14.7)	28 (9.2)	27 (37.5)	< 0.001
COA/IAA	29 (7.7)	17 (5.6)	12 (16.7)	0.002
Shone	6 (1.6)	2 (0.7)	4 (5.6)	0.003
Others^c^	90 (24.0)	69 (22.8)	21 (29.2)	0.253
Echocardiographic characteristics				
LVEDDI (mm)	35.0 (31.0, 39.2)	31.0 (30.8, 38.3)	39.0 (34.3, 45.8)	< 0.001
LVEF (%)	69.0 (65.0, 72.0)	69.5 (65.0, 73.0)	68.0 (65.0, 70.0)	0.101
LVOTG (mmHg)	7.8 (5.8, 38.4)	6.8 (4.8, 19.4)	56.3 (23.5, 94.1)	< 0.001
AR grade ≥ 2	29 (7.7)	5 (1.7)	24 (33.3)	< 0.001
Surgical characteristics				
Histomorphologic type				
Membrane	311 (82.9)	263 (86.8)	47 (65.3)	< 0.001
Fibromuscular ridge	64 (17.1)	40 (13.2)	24 (34.7)	< 0.001
Geometric configuration type				
Circumferential type	74 (19.8)	46 (15.1)	28 (38.9)	< 0.001
Crescent-shaped type	301 (80.2)	257 (84.8)	44 (61.1)	< 0.001
Membrane diameter (mm)	4.0 (3.0, 6.0)	4.0 (3.0, 5.0)	5.0 (4.0, 6.0)	< 0.001
Attachment of membrane to AV	35 (9.3)	20 (6.6)	15 (20.8)	< 0.001
Procedures				
Membranectomy	363 (96.8)	293 (96.7)	70 (97.2)	0.821
Membranectomy + myotomy/myectomy	41 (10.9)	29 (9.6)	12 (16.7)	0.083
AVP	54 (14.4)	27 (8.9)	27 (37.5)	< 0.001
Number of procedures	1.0 (1.0, 1.0)	1.0 (1.0, 1.0)	1.0 (1.0, 2.0)	< 0.001
Follow-up period (y)	3.4 ± 3.0	3.2 ± 2.7	4.4 ± 4.1	0.642

Data are presented as mean ± standard deviation for continuous variables with normal distribution, median (interquartile range) for continuous variables without normal distribution, and *n* (%) for categorical variables. Continuous variables were compared using the Student’s *t* test or Mann–Whitney *U* test, as appropriate; categorical variables were compared with the Chi-square test or Fisher’s exact test

*BSA* body surface area, *NYHA* New York Heart Association, *DSS* discrete subaortic stenosis, *VSD* ventricular septal defect, *PDA* patent ductus arteriosus, *COA/IAA* coarctation/interruption of the aorta, *LVEDDI* left ventricular end-diastolic diameter index, *LVEF* left ventricular ejection fraction, *LVOTG* left ventricular outflow tract gradient, *AR* aortic regurgitation, *AV* aortic valve, *AVP* aortic valvuloplasty, *AVR* aortic valve replacement. ^a^The primary endpoints (+) group comprised patients who met the composite endpoint (cardiac death, AVR/Bentall procedure, LVOTG ≥ 50 mmHg due to recurrence, surgical reintervention, or moderate/severe AV dysfunction); ^b^the primary endpoints (–) group included those who did not experience any endpoint components; ^c^others included congenital heart diseases such as patent foramen ovale, atrial septal defect, persistent left superior vena cava, endocardial cushion defect, double outlet right ventricle, and tetralogy of Fallot

### Follow-up period and endpoints

During follow-up, the pediatric cohort showed the same dominant postoperative pattern as the full cohort, namely recurrent LVOT obstruction and progressive AV dysfunction. In the pediatric cohort, after a mean follow-up of 3.5 years, 72 (19.2%) patients reached the primary endpoint, including 1 death, 9 AV replacements (4 prosthetic AVRs and 5 Ross procedures), 37 patients developed moderate to severe AR or AS. Thirty-three patients required surgical reintervention due to recurrent membrane formation, including 16 for left ventricular outflow tract obstruction and 17 for AV dysfunction. Regarding the secondary endpoints, 50 (13.3%) patients experienced AV dysfunction, comprising 9 AVRs (including 5 Ross procedures), 41 cases of moderate to severe AR and 9 cases of concomitant moderate to severe AS and AR.

### Predictors of the primary and secondary endpoints

**Fig. 2:**
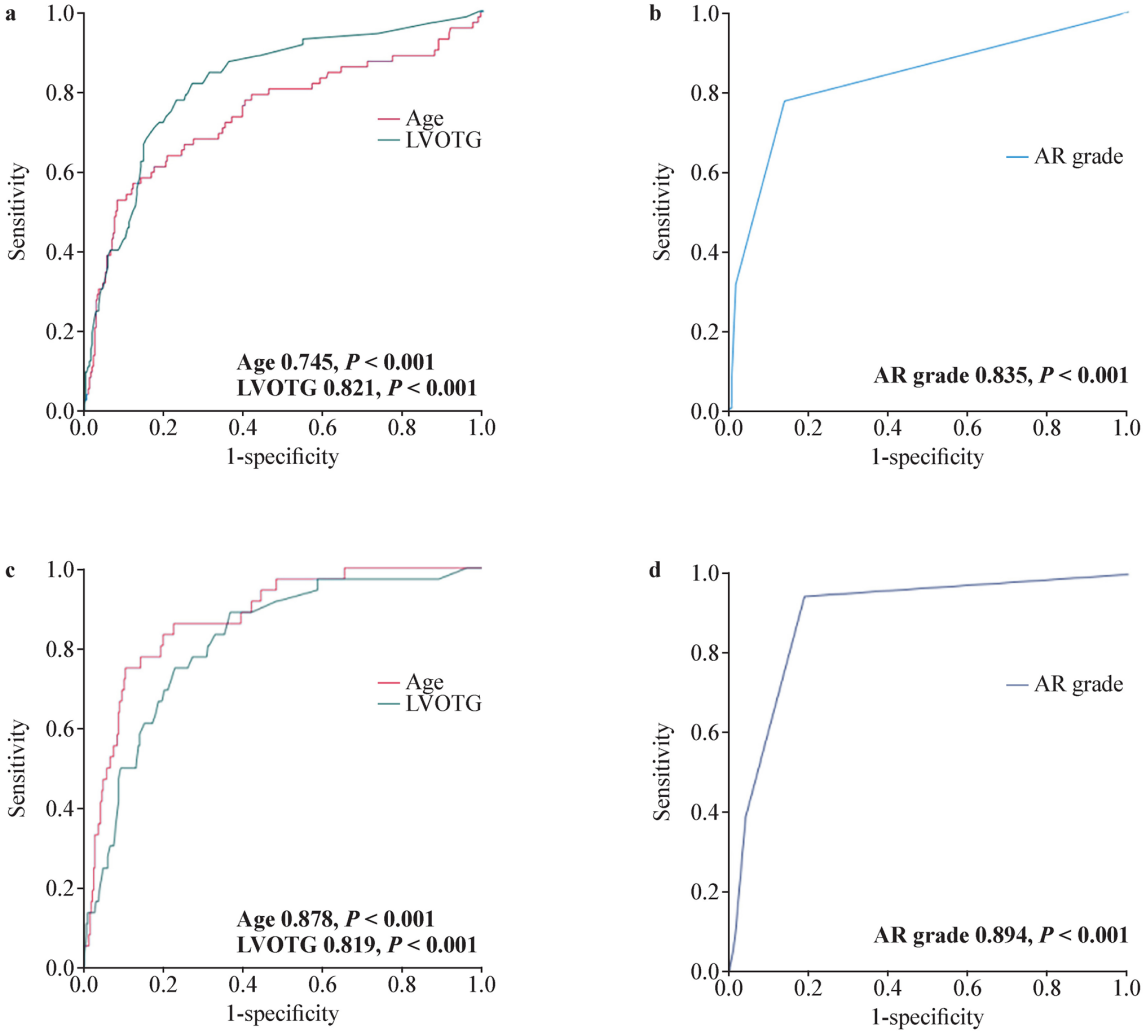
ROC analysis for different endpoints (age < 18 years). The primary endpoint analysis for age and LVOTG (**a**) and for AR grade (**b**). The secondary endpoint analysis for age and LVOTG (**c**) and for AR grade (**d**). Diagonal segments are produced by ties. *ROC* receiver operating characteristic, *LVOTG* left ventricular outflow tract gradient, *AR* aortic regurgitation

ROC analysis in the pediatric cohort demonstrated that LVOTG and age at initial operation were significant predictors of the primary endpoint, with AUCs of 0.821 (95% CI: 0.765–0.877, *P* < 0.001) and 0.745 (95% CI: 0.671–0.818, *P* < 0.001), respectively (Fig. 2a). The optimal cutoff values were identified as 25.8 mmHg for LVOTG and 8.05 years for age, yielding a sensitivity of 78.7% and specificity of 75.7% for LVOTG and a sensitivity of 83.7% and specificity of 79.1% for age. In addition, AR grade was also predictive of the primary endpoint, with an AUC of 0.835 (95% CI: 0.774–0.895, *P* < 0.001) (Fig. 2b). These three parameters were likewise significant predictors of AV dysfunction, with AUCs of 0.878 (95% CI: 0.823–0.933, *P* = 0.027) for age at operation, 0.819 (95% CI: 0.751–0.887, *P* = 0.023) for LVOTG, and 0.894 (95% CI: 0.843–0.945, *P* < 0.001) for AR grade (Fig. 2c, d).

### Cox proportional hazards model and survival analysis

**Fig. 3:**
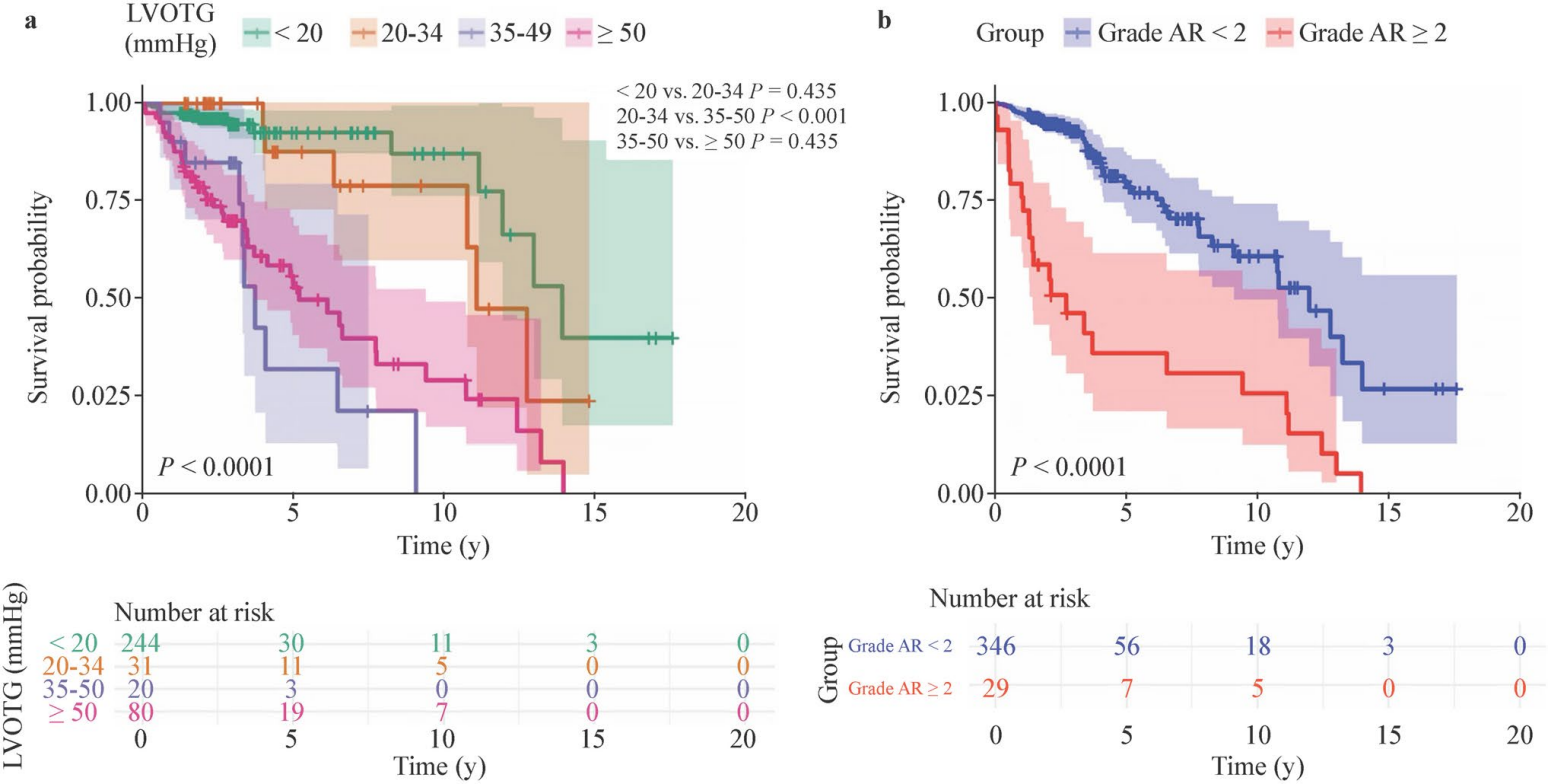
Kaplan–Meier survival curves for the primary endpoint (age < 18 years). Stratified analysis by LVOTG (**a**) and AR grade (**b**). The y-axis represents survival probability, and the x-axis indicates time in years. The shaded areas around the curves represent the 95% confidence intervals. *LVOTG* left ventricular outflow tract gradient, *AR* aortic regurgitation

To evaluate the prognostic value of LVOTG for the primary endpoint, we constructed three separate multivariable Cox regression models using LVOTG thresholds of ≥ 20 mmHg, ≥ 35 mmHg and ≥ 50 mmHg as binary variables (Table 2). After adjusting for potential confounders, all three thresholds were independently associated with the occurrence of adverse events: LVOTG ≥ 20 mmHg (HR = 4.0, 95% CI: 2.0–7.8, *P* < 0.001), LVOTG ≥ 35 mmHg (HR = 6.1, 95% CI: 3.2–11.6, *P* < 0.001) and LVOTG ≥ 50 mmHg (HR = 3.5, 95% CI: 1.9–6.4, *P* < 0.001) (Table 2). The global test of proportional hazards assumption was not significant (*P* = 0.180). Schoenfeld residual plots showed no systematic trends, indicating that the assumption was satisfied. At 3 years, AUCs for LVOTG ≥ 20, ≥ 35 and ≥ 50 mmHg were 0.82 (95% CI: 0.76–0.88), 0.78 (95% CI: 0.72–0.85) and 0.77 (95% CI: 0.70–0.83), respectively. Pairwise comparisons were conducted to evaluate the differences in ΔAUC between LVOTG thresholds. The difference between the ≥ 20 mmHg and ≥ 35 mmHg groups was statistically significant (ΔAUC = 0.05; 95% CI: 0.01–0.08; raw *P* = 0.006; Holm-adjusted *P* = 0.012). Similarly, the difference between the ≥ 20 mmHg and ≥ 50 mmHg groups was significant (ΔAUC = 0.06; 95% CI: 0.01–0.09; raw *P* = 0.011; Holm-adjusted *P* = 0.018). In contrast, the difference between the ≥ 35 mmHg and ≥ 50 mmHg groups was not statistically significant (ΔAUC = 0.01; raw *P* = 0.571; Holm-adjusted *P* = 0.761). Results were consistent at 1 and 5 years and with Uno’s C-index. Kaplan–Meier survival analysis further demonstrated a significant overall difference in event-free survival among patients stratified by LVOTG categories (< 20, 20–34, 35–49, and ≥ 50 mmHg; *P* < 0.001), whereas no significant difference was observed between the 35–49 mmHg and ≥ 50 mmHg groups (Fig. 3a). In addition, patients with AR grade ≥ 2 had significantly worse event-free survival than those with none or mild AR (Fig. 3b, *P* < 0.001). These results indicate that an LVOTG threshold of 35 mmHg confers a risk comparable to the current 50 mmHg standard while providing superior discriminatory ability over the 20 mmHg cutoff.

**Table 2:** Multivariable Cox regression models for the primary endpoint based on LVOTG thresholds in pediatric patients (age < 18 years)

Variables	Model 1 (LVOTP ≥ 20 mmHg)		Model 2 (LVOTP ≥ 35 mmHg)		Model 3 (LVOTP ≥ 50 mmHg)	
	HR (95% CI)	*P*	HR (95% CI)	*P*	HR (95% CI)	*P*
Age	1.1 (1.0, 1.2)	< 0.001	1.1 (1.1, 1.2)	< 0.001	1.1 (1.1, 1.2)	< 0.001
AR grade ≥ 2	2.6 (1.4, 4.6)	0.002	2.0 (1.1, 3.5)	0.018	2.0 (1.1, 3.6)	0.019
Shone’s complex	1.4 (0.5, 4.2)	0.551	0.9 (0.3, 2.8)	0.891	1.2 (0.4, 3.5)	0.796
Prior surgery^a^		0.250		0.705		0.247
Prior non-DSS surgery	1.7 (0.9, 3.0)	0.098	1.2 (0.7, 2.3)	0.472	1.6 (0.9, 2.9)	0.111
Prior DSS-related surgery	1.1 (0.5, 2.6)	0.817	0.9 (0.4, 2.1)	0.810	0.9 (0.4, 2.2)	0.898
AVP	1.5 (0.9, 2.6)	0.143	2.2 (1.3, 3.7)	0.004	1.9 (1.1, 3.3)	0.017
Membrane diameter	1.1 (1.0, 1.2)	0.161	1.1 (1.0, 1.2)	0.137	1.0 (0.9, 1.2)	0.369
Membrane subtype^b^	1.0 (0.6, 1.6)	0.904	1.5 (0.9, 2.6)	0.157	1.3 (0.7, 2.3)	0.412
Attachment of membrane to AV	1.1 (0.6, 2.1)	0.741	0.9 (0.5, 1.7)	0.806	1.0 (0.5, 1.9)	0.995
LVOTG	4.0 (2.0, 7.8)	< 0.001	6.1 (3.2, 11.6)	< 0.001	3.5 (1.9, 6.4)	< 0.001

*HR* hazard ratio, *CI* confidence interval, *LVOTG* left ventricular outflow tract gradient, *AR* aortic regurgitation, *DSS* discrete subaortic stenosis, *AVP* aortic valvuloplasty, *AV* aortic valve

^a^Prior surgery is a combined variable, and therefore no specific HR value is provided. The hazard ratios are instead reported separately in the following two subcategories, namely prior non-DSS surgery and prior DSS-related surgery; ^b^Membrane subtype refers to the two morphological classifications of DSS: circumferential membrane and crescent-shaped membrane

For the secondary endpoint of AV dysfunction, consistent multivariable Cox regression models were constructed using the same LVOTG thresholds and covariate adjustments (Table 3). All three thresholds were independently associated with an increased risk of AV dysfunction: LVOTG ≥ 20 mmHg (HR = 2.9, 95% CI: 1.1–7.4, *P* = 0.027), LVOTG ≥ 35 mmHg (HR = 6.2, 95% CI: 2.5–15.4, *P* < 0.001) and LVOTG ≥ 50 mmHg (HR = 6.8, 95% CI: 2.9–16.2, *P* < 0.001). Of note, although all three LVOTG thresholds were predictive, the incremental risk between 35 and 50 mmHg was modest, further supporting the clinical relevance of 35 mmHg as a potential intervention threshold. Kaplan–Meier survival curves further confirmed a significant difference in AV dysfunction-free survival between patients with AR grade ≥ 2 and those with none or mild AR. In the overall cohort, the predictive value of LVOTG for both primary and secondary endpoints was consistent with the findings observed in the pediatric cohort (Supplementary Tables 2, 3, 4 and Supplementary Fig. 2). The distribution of baseline variables varied significantly across different LVOTG severity groups (Supplementary Table 5**)**.

**Table 3:** Multivariable Cox regression models for the second endpoint based on LVOTG thresholds in pediatric patients (age < 18 years)

Variables	Model 1 (LVOTP ≥ 20 mmHg)		Model 2 (LVOTP ≥ 35 mmHg)		Model 3 (LVOTP ≥ 50 mmHg)	
	HR (95% CI)	*P*	HR (95% CI)	*P*	HR (95% CI)	*P*
Age	1.2 (1.1, 1.3)	< 0.001	1.2 (1.2, 1.3)	< 0.001	1.3 (1.2, 1.4)	< 0.001
AR grade ≥ 2	3.0 (1.3, 7.0)	0.009	2.3 (1.0, 5.2)	0.040	3.2 (1.4, 7.2)	0.006
Shone’s complex	0.0 (0.0, 0.0)	0.977	0.0 (0.0, 0.0)	0.983	0.0 (0.0, 0.0)	0.981
Prior surgery^a^		0.041		0.098		0.025
Prior non-DSS surgery	1.9 (0.9, 4.0)	0.094	1.5 (0.7, 3.1)	0.338	1.9 (0.9, 4.0)	0.083
Prior DSS-related surgery	0.2 (0.0, 1.4)	0.102	0.1 (0.0, 1.2)	0.072	0.1 (0.0, 1.1)	0.062
AVP	1.6 (0.7, 3.3)	0.247	2.1 (1.0, 4.4)	0.042	1.5 (0.7, 3.3)	0.267
Membrane diameter	1.1 (1.0, 1.3)	0.072	1.1 (1.0, 1.3)	0.038	1.1 (1.0, 1.3)	0.104
Membrane subtype^b^	1.6 (0.7, 3.5)	0.272	2.4 (1.1, 5.6)	0.037	3.4 (1.4, 8.2)	0.007
Attachment of membrane to AV	1.6 (0.6, 4.0)	0.351	1.2 (0.5, 3.1)	0.635	1.1 (0.4, 2.8)	0.821
LVOTG	2.9 (1.1, 7.4)	0.027	6.2 (2.5, 15.4)	< 0.001	6.8 (2.9, 16.2)	< 0.001

*HR* hazard ratio, *CI* confidence interval, *LVOTG* left ventricular outflow tract gradient, *AR* aortic regurgitation, *DSS* discrete subaortic stenosis, *AVP* aortic valvuloplasty, *AV* aortic valve

^a^Prior surgery is a combined variable, and therefore no specific HR value is provided. The hazard ratios are instead reported separately in the following two subcategories, namely prior non-DSS surgery and prior DSS-related surgery; ^b^Membrane subtype refers to the two morphological classifications of DSS: circumferential membrane and crescent-shaped membrane

## Discussion

This retrospective cohort study examined postoperative outcomes in a large series of patients with DSS who underwent surgical intervention. Because DSS is predominantly a pediatric disease, the present results should be interpreted primarily from the pediatric perspective. Our findings demonstrate that elevated LVOTG and moderate to severe AR are important predictors of adverse clinical outcomes, and that an LVOTG threshold of 35 mmHg provided prognostic utility comparable to the widely accepted cutoff of 50 mmHg. These data suggest that current surgical indications may warrant further evaluation in future studies toward earlier intervention in children before irreversible aortic valve injury becomes established.

In children, DSS is not merely a fixed anatomical obstruction. With ongoing somatic growth, abnormal LVOT flow acceleration and turbulence may continue to affect the AV and sub-valvular region, contributing to membrane progression, recurrent obstruction and worsening AR over time. This pediatric pathophysiological background helps explain why postoperative recurrence and AV dysfunction remained common in our cohort even though surgery was performed according to currently recognized indications [2, 7, 10]. Unlike previous studies [11, 12], we included cases in which the native AV could not be preserved and required AVR or a Bentall procedure as part of the AV dysfunction category. This broader definition may account for the higher incidence of adverse outcomes reported in our study, but we believe it better reflects clinically meaningful long-term pediatric burden, because loss of the native AV has major implications for growth, repeat intervention and lifelong management.

Timing is particularly important in pediatric DSS. Delayed intervention may allow a prolonged period of abnormal hemodynamic stress during childhood, when the LVOT and aortic valve are still developing, thereby increasing the likelihood of fixed valve injury that cannot be fully reversed by membrane resection alone. The current recommended surgical indication of LVOTP ≥ 50 mmHg has been based largely on limited empirical evidence [10, 11, 13–15]. In the present study, ROC analysis identified low gradient and age cutoffs associated with adverse outcomes, and multivariable Cox regression showed that LVOTG thresholds of ≥ 20 mmHg, ≥ 35 mmHg and ≥ 50 mmHg were all independently associated with increased risk of both endpoints. However, pairwise comparisons revealed no significant difference in risk between LVOTG ≥ 35 mmHg and ≥ 50 mmHg, while both were associated with a significantly higher risk compared with the ≥ 20 mmHg threshold. This finding suggests that a threshold of 35 mmHg may offer a more practical pediatric risk-alert threshold, improving sensitivity for identifying children at risk while maintaining discrimination comparable to the traditional 50 mmHg cutoff. In parallel, older age at surgery and preoperative AR grade ≥ 2 consistently emerged as markers of worse prognosis, supporting the concept that surgical timing should be integrated with growth-related valve vulnerability rather than determined by gradient alone.

The pediatric-specific findings also strengthen the clinical importance of long-term follow-up. Even after apparently successful resection, children remain at risk of recurrent membrane formation, progressive AR and future reintervention across a long-life course. In our cohort, some patients underwent surgery primarily for associated cardiac lesions (e.g., VSD), with concomitant subaortic membrane resection performed during the same procedure. These patients therefore underwent earlier DSS intervention, had lower preoperative LVOT gradients and experienced fewer DSS-related adverse outcomes. Importantly, the study endpoints were defined according to DSS-related events, and outcomes attributable solely to repair of associated lesions were excluded, allowing this subgroup to function as an internal clinical comparison rather than a source of bias. These real-world observations further support the view that waiting for a very high LVOT gradient may miss the optimal window for preserving native aortic valve function in pediatric patients.

From a surgical planning perspective, our findings support a more nuanced pediatric decision-making process that incorporates not only LVOTG but also anatomical features such as valve involvement and membrane characteristics [14, 16–18]. Children with moderate gradients (e.g. ≥ 35 mmHg), concomitant AR or membrane adherence to the AV may derive particular benefit from earlier intervention. Taken together, our results emphasize that pediatric DSS management should aim not only to relieve obstruction at the time of surgery, but also to minimize cumulative valve injury, reduce the need for repeat procedures and preserve long-term cardiac development.

Several limitations of this study should be acknowledged. First, the retrospective design inherently carries risks of selection bias and unmeasured confounding. Second, all data were collected from a single high-volume tertiary cardiac center, which may limit the generalizability of our findings to other centers with differing surgical practices or patient demographics. Third, although echocardiographic measurements were reviewed from archived digital images, inter-observer variability in LVOT gradient assessment may still exist. Fourth, the follow-up period, while adequate for short- to mid-term outcomes, may not fully capture long-term complications such as progressive AV dysfunction or late reoperation. In addition, although revised LVOTG thresholds were proposed based on multivariable analysis and ROC curves, external validation in a prospective cohort is necessary before incorporation into clinical guidelines.

In conclusion, the surgical prognosis of DSS remains suboptimal, primarily due to the high recurrence rate and the risk of progressive AV dysfunction. LVOTG ≥ 35 mmHg was independently associated with adverse outcomes, with predictive value comparable to the traditional 50 mmHg threshold.

## Supplementary Information

Below is the link to the electronic supplementary material.

Supplementary file1 (PDF 566 KB)

## Data Availability

The data that support the findings of this study are not publicly available due to reasons of sensitivity but are available from the corresponding author upon reasonable request.
