# Supplementary material for: Discrete subaortic stenosis: prognostic value of the left ventricular outflow tract gradient and implications for earlier surgical intervention
Source: World J Pediatr. 2026 Jun 11;22(5):586–95. doi: 10.1007/s12519-026-01048-z (PMC13290889; doi:10.1007/s12519-026-01048-z)
Supplement: Supplementary file 1 — Supplementary file1 (PDF 566 KB) [file 12519_2026_1048_MOESM1_ESM.pdf]

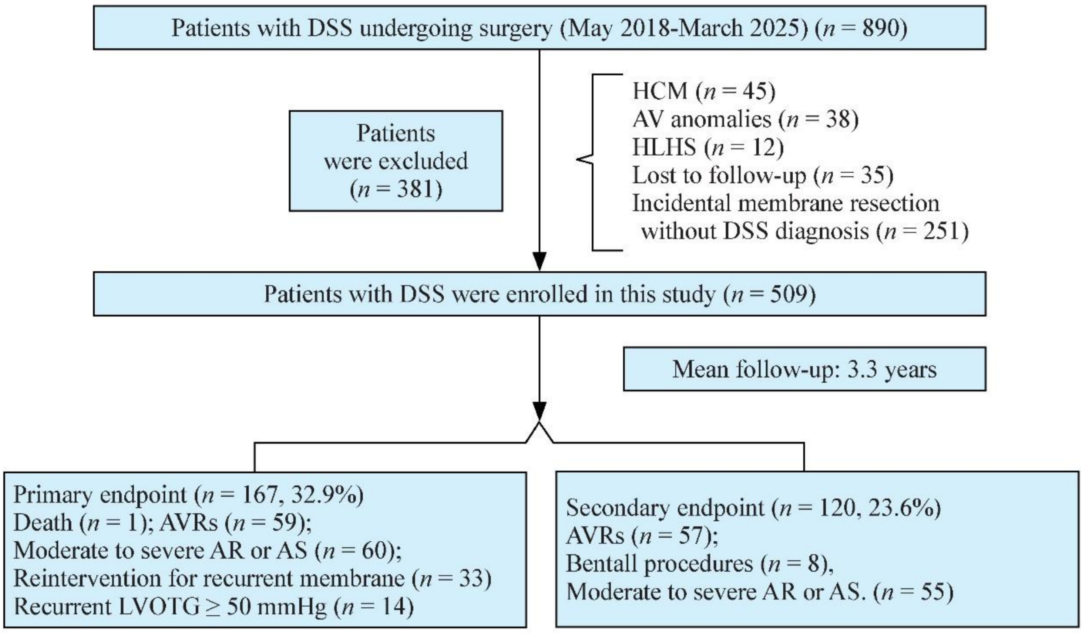

Supplementary Fig. 1 Patient selection process. *DSS* discrete subaortic stenosis, *HCM* hypertrophic cardiomyopathy, *AV* aortic valve, *HLHS* hypoplastic left heart syndrome, *AVR* aortic valve replacement, *AR* aortic regurgitation, *AS* aortic stenosis, *LVOTG* left ventricular outflow tract gradients.

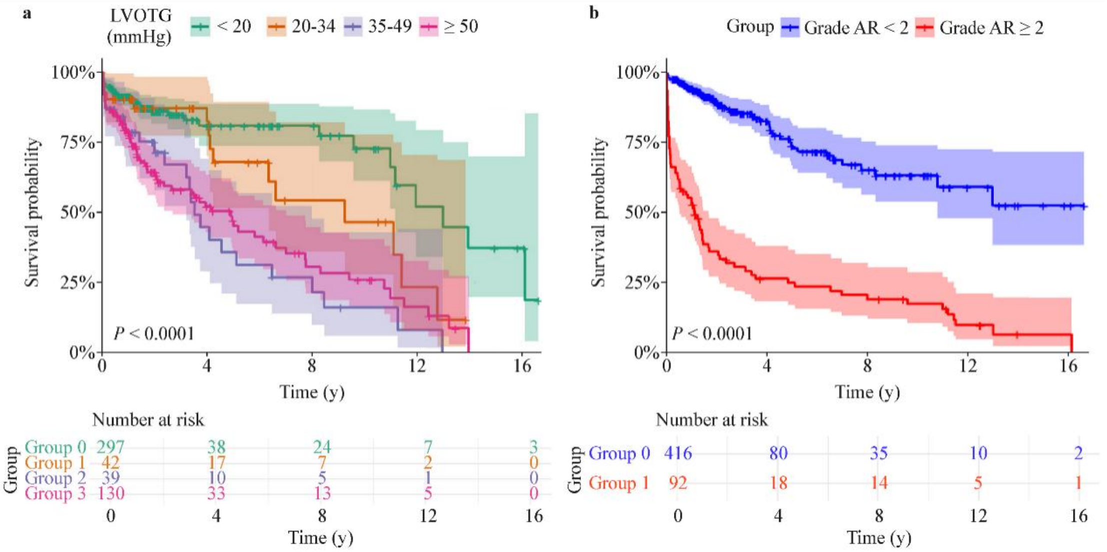

Supplementary Fig. 2 Kaplan–Meier survival curves for AV dysfunction (the entire cohort). (a) Stratified by different levels of LVOT pressure. (b) Stratified by aortic regurgitation grade. The y-axis represents survival probability, and the x-axis indicates time in years. The shaded areas around the curves represent the 95% confidence intervals. *AV* aortic valve, *LVOTG* left ventricular outflow tract gradients, *AR* aortic

regurgitation.

Supplementary Table 1 Baseline characteristics according to primary endpoint (the entire cohort).

Variables	General $N = 508$	Primary endpoint (-) [b] $n = 341$	Primary endpoint (+) [a] $n = 167$	P
**Clinical characteristics**				
Age (y)	5.1 (1.8, 20.4)	3.3 (1.3, 6.3)	25.7 (9.6, 50.6)	< 0.001
Male	272 (53.5)	187 (54.8)	85 (50.9)	0.403
BSA (m^2)	0.8 (0.5, 1.5)	0.6 (0.4, 0.9)	1.5 (1.1, 1.7)	< 0.001
NYHA class ≥ II	151 (29.7)	57 (16.7)	94 (56.3)	< 0.001
History of cardiac procedures	79 (15.6)	29 (8.5)	50 (29.9)	< 0.001
History of DSS-related procedures	14 (2.8)	2 (0.6)	12 (7.2)	< 0.001
**Concomitant cardiac malformations**				
VSD	298 (58.7)	253 (74.2)	45 (26.9)	< 0.001
PDA	68 (13.4)	32 (9.4)	36 (21.6)	< 0.001
COA/IAA	31 (6.1)	18 (5.3)	13 (7.8)	0.268
Shone's complex	7 (1.4)	3 (0.9)	4 (2.4)	0.169
Others[c]	114 (22.4)	76 (22.3)	38 (22.8)	0.906
**Echocardiographic characteristics**				
LVEDDI (mm/m^2)	38.0 (32.0, 46.0)	35.0 (31.0, 40.0)	46.0 (39.0, 55.0)	< 0.001
LVEF (%)	68.0 (64.0, 71.0)	69.0 (65.0, 72.0)	66.0 (61.0, 70.0)	< 0.001
LVOTG (mmHg)	11.6 (5.8, 51.8)	6.8 (5.8, 21.2)	46.2 (19.4, 88.4)	< 0.001
AR grade ≥ 2	92 (18.1)	10 (2.9)	82 (49.1)	< 0.001
**Surgical characteristics**				
**Histomorphologic type**				
Membrane	296 (58.3)	205 (60.2)	91 (54.5)	0.381
Fibromuscular ridge	212 (41.7)	136 (39.8)	76 (45.5)	0.002

**Geometric configuration type**				
Circumferential membrane	110 (21.7)	54 (15.8)	56 (33.5)	< 0.001
Crescent-shaped membrane	398 (78.3)	287 (84.2)	111 (66.5)	< 0.001
Membrane diameter (mm)	5.0 (3.0, 6.0)	4.0 (3.0, 5.0)	5.0 (4.0, 7.0)	< 0.001
Attachment of membrane to AV	52 (10.2)	24 (7.0)	28 (16.8)	< 0.001
**Procedures**				
Membranectomy	482 (94.9)	328 (96.2)	154 (92.2)	0.056
Membranectomy + myotomy/myectomy	60 (11.8)	34 (10)	26 (15.6)	0.066
AVP	66 (13.0)	30 (8.8)	36 (21.6)	< 0.001
Number of procedures	1.1 ± 0.3	1.0 ± 0.2	1.2 ± 0.4	< 0.001
**Follow-up period (y)**	3.3 ± 3.2	3.3 ± 2.3	3.3 ± 2.8	0.782

Data are presented as mean ± standard deviation for continuous variables with normal distribution, median (interquartile range) for continuous variables without normal distribution, and *n* (%) for categorical variables. Continuous variables were compared using the Student's *t* test or Mann–Whitney *U* test, as appropriate; categorical variables were compared with the Chi-square test or Fisher's exact test. *BSA* body surface area, *NYHA* New York Heart Association, *DSS* discrete subaortic stenosis, *VSD* ventricular septal defect, *PDA* patent ductus arteriosus, *COA/IAA* coarctation/interruption of the aorta, *LVEDDI* left ventricular end-diastolic diameter index, *LVEF* left ventricular ejection fraction, *LVOTG* left ventricular outflow tract gradient, *AR* aortic regurgitation, *AV* aortic valve, *AVP* aortic valvuloplasty, *AVR* aortic valve replacement. [a]The primary endpoints (+) group comprised patients who met the composite endpoint (cardiac death, AVR/Bentall procedure, LVOTG ≥ 50 mmHg due to recurrence, surgical reintervention, or moderate/severe AV dysfunction); [b]the Primary endpoints (–) group included those who did not experience any endpoint components; [c]others include congenital heart diseases such as patent foramen ovale, atrial septal defect, persistent left superior vena cava, endocardial cushion defect, double outlet right ventricle, and tetralogy of Fallot

Supplementary Table 2 Baseline characteristics according to aortic valve dysfunction occurrence (the entire cohort).

Variables	general *n* = 508	AV dysfunction (-)[b] *n* = 120	AV dysfunction (+)[a] *n* = 388	*P*
**Clinical characteristics**				
Age (y)	5.1 (1.8, 20.4)	3.5 (1.3, 7.5)	35.4 (10.1, 55.9)	< 0.001
Male	272 (53.5)	212 (54.6)	60 (50.0)	0.373
BSA (m^2)	0.8 (0.5, 1.5)	0.64 (0.4, 1.0)	1.6 (1.4, 1.7)	< 0.001
NYHA class ≥II (m^2)	151 (29.7)	67 (17.3)	84 (70.0)	< 0.001
history of procedure	79 (15.6)	46 (11.9)	33 (27.5)	< 0.001

history of DSS-related procedure	14 (2.8)	10 (2.9)	4 (2.4)	0.09
**concomitant malformations**				
VSD	298 (58.7)	266 (68.6)	32 (26.7)	< 0.001
PDA	68 (13.4)	46 (11.9)	22 (18.3)	0.069
COA/IAA	31 (6.1)	24 (6.2)	7 (5.8)	0.888
Shone	7 (1.4)	7 (1.8)	0 (0)	0.138
Others[c]	114 (22.4)	91 (23.5)	23 (19.2)	0.325
**Echocardiography characteristics**				
LVEDDI (mm)	38.0 (32.0, 46.0)	36.0 (31.0, 41.0)	50.0 (44.0, 58.0)	< 0.001
LVEF (%)	68.0 (64.0, 71.0)	69.0 (65.0, 72.0)	65.0 (60.0, 68.8)	< 0.001
LVOTG (mmHg)	11.6 (5.8, 51.8)	7.8 (5.8, 33.6)	46.2 (17.6, 87.4)	< 0.001
AR grade ≥ 2	92 (18.1)	23 (5.9)	69 (57.5)	< 0.001
**Surgical Characteristics**				
**Histomorphologic type**				
membrane	396			
fibromuscular ridge	113			
**Geometric configuration type**				
circumferential membrane	110 (21.7)	72 (18.6)	38 (31.7)	0.002
crescent-shaped membrane	398 (78.3)	316 (81.4)	82 (68.3)	0.002
membrane diameter (mm)	5.0 (3.0, 6.0)	4.0 (3.0, 6.0)	5.0 (4.0, 7.0)	< 0.001
Attachment of membrane to aortic valve	52 (10.2)	33 (8.5)	19 (15.8)	0.021
**procedures**				
Membranectomy	482 (94.9)	372 (95.9)	110 (91.7)	0.067
Membranectomy+myotomy	60 (11.8)	44 (11.3)	16 (13.3)	0.554
AVP	66 (13)	43 (11.1)	23 (19.2)	0.021
number of procedures	1.1 ± 0.3	1.1 ± 0.3	1.1 ± 0.3	0.644
**Follow-up duration (y)**	1.3 (0.8, 3.0)	1.4 (0.9, 2.9)	0.9 (0.1, 3.3)	< 0.001

Data are presented as mean ± standard deviation for continuous variables with normal distribution, median (interquartile range) for continuous variables without normal distribution, and *n* (%) for categorical variables. Continuous variables were compared using the Student's *t* test or Mann–Whitney *U* test, as appropriate; categorical variables were compared with the Chi-square test or Fisher's exact test. *BSA* body surface area, *NYHA* New York Heart Association, *DSS* discrete subaortic stenosis, *VSD* ventricular septal defect, *PDA* patent ductus arteriosus, *COA/IAA* coarctation/interruption of the aorta, *LVEDDI* left ventricular end-diastolic diameter index, *LVEF* left ventricular ejection fraction, *LVOTG* left ventricular outflow tract gradient, *AR* aortic regurgitation, *AV* aortic valve, *AVP* aortic valvuloplasty, *AVR* aortic valve replacement. [a]The primary endpoints (+) group comprised patients who met the composite endpoint (cardiac death, AVR/Bentall procedure, LVOTG ≥ 50 mmHg due to recurrence, surgical reintervention, or moderate/severe AV dysfunction); [b]the Primary endpoints (–) group included

those who did not experience any endpoint components; [c]others include congenital heart diseases such as patent foramen ovale, atrial septal defect, persistent left superior vena cava, endocardial cushion defect, double outlet right ventricle, and tetralogy of Fallot

Supplementary Table 3 Multivariable cox regression models for the primary endpoint based on LVOTG thresholds (the entire cohort).

Variables	Model 1 (LVOTG ≥ 20 mmHg)		Model 2 (LVOTG ≥ 35 mmHg)		Model 3 (LVOTG ≥ 50 mmHg)	
	HR (95% CI)	*P*	HR (95% CI)	*P*	HR (95% CI)	*P*
Age	1.01 (1.01, 1.02)	< 0.001	1.01 (1.01, 1.02)	< 0.001	1.02 (1.01, 1.02)	< 0.001
AR grade ≥ 2	3.03 (2.12, 4.34)	< 0.001	3.18 (2.23, 4.54)	< 0.001	3.24 (2.26, 4.64)	< 0.001
Shone's complex	1.57 (0.57, 4.33)	0.387	1.46 (0.53, 4.06)	0.468	1.70 (0.61, 4.77)	0.310
Prior surgery[a]		0.447		0.229		0.196
Prior non-DSS surgery	1.25 (0.84, 1.87)	0.267	1.35 (0.91, 2.00)	0.141	1.37 (0.92, 2.04)	0.123
Prior DSS-related surgery	0.88 (0.46, 1.68)	0.692	0.82 (0.43, 1.57)	0.546	0.81 (0.42, 1.55)	0.518
AVP	1.00 (0.66, 1.51)	0.991	1.05 (0.69, 1.58)	0.829	1.07 (0.71, 1.62)	0.739
Membrane diameter	0.98 (0.94, 1.02)	0.371	0.99 (0.95, 1.03)	0.541	0.99 (0.95, 1.03)	0.500
Membrane subtype[b]	0.91 (0.60, 1.41)	0.683	0.83 (0.54, 1.29)	0.408	0.90 (0.58, 1.39)	0.63
Attachment of membrane to AV	0.98 (0.68, 1.41)	0.906	0.99 (0.68, 1.44)	0.960	0.85 (0.59, 1.24)	0.405
LVOTG	2.30 (1.58, 3.35)	< 0.001	2.23 (1.57, 3.18)	< 0.001	1.53 (1.07, 2.19)	0.020

HR hazard ratio, *CI* confidence interval, *LVOTG* left ventricular outflow tract gradient, *AR* aortic regurgitation, *DSS* discrete subaortic stenosis, *AVP* aortic valvuloplasty, *AV* aortic valve. [a]Prior surgery is a combined variable, and therefore no specific HR value is provided. The hazard ratios are instead reported separately in the following two subcategories, namely prior non-DSS surgery and prior DSS-related surgery; [b]membrane subtype refers to the two morphological classifications of DSS: circumferential membrane and crescent-shaped membrane

Supplementary Table 4 Multivariable cox regression models for aortic valve

dysfunction based on LVOTG thresholds (the entire cohort).

Variables	model1 (LVOTG ≥ 20mmHg)		model2 (LVOTG ≥35mmHg)		model3 (LVOTG ≥ 50mmHg)	
	HR (95% CI)	*P*	HR (95% CI)	*P*	HR (95% CI)	*P*
age	1.01 (1.01, 1.02)	< 0.001	1.01 (1.01, 1.02)	< 0.001	1.02 (1.01, 1.02)	< 0.001
AR grade⩾ 2	3.03 (2.12, 4.34)	< 0.001	3.18 (2.23, 4.54)	< 0.001	3.24 (2.26, 4.64)	< 0.001
Shone's complex	1.57 (0.57, 4.33)	0.387	1.46 (0.53, 4.06)	0.468	1.70 (0.61, 4.77)	0.31
prior surgery[a]		0.447		0.229		0.196
Prior non–DSS surgery	1.25 (0.84, 1.87)	0.267	1.35 (0.91, 2.00)	0.141	1.37 (0.92, 2.04)	0.123
Prior DSS related surgery	0.88 (0.46, 1.68)	0.692	0.82 (0.43, 1.57)	0.546	0.81 (0.42, 1.55)	0.518
AVP	1.00 (0.66, 1.51)	0.991	1.05 (0.69, 1.58)	0.829	1.07 (0.71, 1.62)	0.739
membrane diameter (mm)	0.98 (0.94, 1.02)	0.371	0.99 (0.95, 1.03)	0.541	0.99 (0.95, 1.03)	0.5
membrane subtype[b]	0.91 (0.60, 1.41)	0.683	0.83 (0.54, 1.29)	0.408	0.90 (0.58, 1.39)	0.63
Attachment of membrane to AV	0.98 (0.68, 1.41)	0.906	0.99 (0.68, 1.44)	0.96	0.85 (0.59, 1.24)	0.405
LVOTG	2.30 (1.58, 3.35)	< 0.001	2.23 (1.57, 3.18)	< 0.001	1.53 (1.07, 2.19)	0.02

HR hazard ratio, *CI* confidence interval, *LVOTG* left ventricular outflow tract gradient, *AR* aortic regurgitation, *DSS* discrete subaortic stenosis, *AVP* aortic valvuloplasty, *AV* aortic valve. [a]Prior surgery is a combined variable, and therefore no specific HR value is provided. The hazard ratios are instead reported separately in the following two subcategories, namely prior non-DSS surgery and prior DSS-related surgery; [b]membrane subtype refers to the two morphological classifications of DSS: circumferential membrane and crescent-shaped membrane

Supplementary Table 5 Baseline characteristics stratified according to the different level of LVOTG (the entire cohort).

Variables	general *n* = 508	LVOTG < 20 (*n* = 297)	LVOTG 20-34 (*n* = 42)	LVOTG 35-50 (*n* = 39)	LVOTG ⩾ 50 (*n* =130)	*P*
**Clinical characteristics**						

age (y)	5.1 (1.8, 20.4)	3.3 (1.2, 8.0)	6.4 (2.0, 23.1)	17.7 (4.5, 48.6)	11.2 (5.1, 44.3)	< 0.001
Male	272 (53.5)	171 (57.6)	28 (66.7)	18 (46.2)	55 (42.3)	0.006
BSA (m^2)	0.8 (0.5, 1.5)	0.6 (0.4, 1.0)	0.7 (0.5, 1.4)	1.4 (0.8, 1.6)	1.3 (0.8, 1.6)	< 0.001
NYHA class ≥II	151 (29.7)	59 (19.9)	14 (33.3)	14 (35.9)	64 (49.2)	< 0.001
history of procedure	79 (15.6)	29 (8.5)	50 (29.9)	0	0	< 0.001
**Concomitant malformations**						
VSD	298 (58.7)	248 (83.5)	16 (38.1)	10 (25.6)	24 (18.5)	< 0.001
PDA	68 (13.4)	22 (7.4)	8 (19.0)	10 (25.6)	28 (21.5)	< 0.001
COA/IAA	31 (6.1)	12 (4.0)	2 (4.8)	1 (2.6)	16 (12.3)	0.008
Shone	7 (1.4)	0	1 (2.4)	0	6 (1.8)	0.002
others[a]	114 (22.4)	68 (22.9)	12 (28.6)	7 (17.9)	27 (20.8)	0.659
**Echocardiography characteristics**						
LVEDDI (mm)	38.0 (32.0, 46.0)	37.0 (32.0, 43.0)	38.5 (30.8, 49.3)	44.0 (35.0, 52.0)	41.0 (35.0, 47.0)	< 0.001
LVEF (%)	68.0 (64.0, 71.0)	68.0 (64.0, 71.0)	68.0 (63.0, 71.0)	68.0 (63.0, 70.0)	68.0 (63.8, 73.0)	0.723
LVOTG (mmHg)	11.6 (5.8, 51.8)	5.8 (4.8, 9.0)	27.0 (22.6, 31.4)	41.0 (38.4, 46.2)	81.0 (70.6, 104.0)	< 0.001
AR grade ≥ 2	92 (18.1)	33 (11.1)	8 (19.0)	13 (33.3)	38 (29.2)	< 0.001
**Surgical Characteristics**						
**Histomorphologic type**						
membrane	296	205 (60.2)	91 (54.5)	15 (38.5)	58 (44.6)	0.381

fibromuscular ridge	212	136 (39.8)	76 (45.5)	24 (61.5)	72 (55.4)	0.002
**Geometric configuration type**						
circumferential membrane	110 (21.7)	29 (9.8)	6 (14.3)	15 (38.5)	60 (46.2)	< 0.001
crescent-shaped membrane	398 (78.3)	268 (90.2)	36 (85.7)	24 (61.5)	70 (53.8)	< 0.001
membrane diameter (mm)	5.0 (3.0, 6.0)	4.0 (3.0, 5.0)	4.0 (3.0, 6.0)	5.0 (4.0, 6.0)	5.0 (4.0, 7.0)	0.080
Attachment of membrane to AV	52 (10.2)	14 (4.7)	3 (7.1)	7 (17.9)	28 (21.5)	< 0.001
**Procedures**						
Membranectomy	482 (94.9)	281 (94.6)	39 (92.9)	37 (94.9)	125 (96.2)	0.842
Membranectomy+myotomy/myectomy	60 (11.8)	15 (5.1)	5 (11.9)	7 (17.9)	33 (25.4)	< 0.001
AVP	66 (13.0)	17 (5.7)	9 (21.4)	4 (10.3)	36 (27.7)	< 0.001
number of procedures						0.060
1		282 (94.9)	37 (88.1)	36 (92.3)	115 (88.5)	
2		15 (5.1)	4 (9.5)	2 (5.1)	14 (10.8)	
3		0	1 (2.4)	1 (2.6)	1 (0.8)	
**Follow-up duration (y)**	3.3 ± 3.2	3.1 ± 3.1	4.6 ± 3.8	3.4 ± 3.1	3.4 ± 3.5	0.026

Data are presented as mean ± standard deviation for continuous variables with normal distribution, median (interquartile range) for continuous variables without normal distribution, and *n* (%) for categorical variables. Continuous variables were compared using the Student's *t* test or Mann–Whitney *U* test, as appropriate; categorical variables were compared with the Chi-square test or Fisher's exact test. *BSA* body surface area, *NYHA* New York Heart Association, *DSS* discrete subaortic stenosis, *VSD* ventricular septal defect, *PDA* patent ductus arteriosus, *COA/IAA* coarctation/interruption of the aorta, *LVEDDI* left ventricular end-diastolic diameter index, *LVEF* left ventricular ejection fraction, *LVOTG* left ventricular outflow tract gradient, *AR* aortic regurgitation, *AV* aortic valve, *AVP* aortic valvuloplasty, *AVR* aortic valve replacement.[a]others include congenital heart diseases such as patent foramen ovale, atrial septal defect, persistent left superior vena cava, endocardial cushion defect, double outlet right ventricle, and tetralogy of Fallot
